# Supplementary material for: Metabolomics facilitates differential diagnosis in common inherited retinal degenerations by exploring their profiles of serum metabolites
Source: Nat Commun. 2024 Apr 26;15:3562. doi: 10.1038/s41467-024-47911-3 (PMC11053129; doi:10.1038/s41467-024-47911-3)
Supplement: Supplementary file 1 — Supplementary information [file 41467_2024_47911_MOESM1_ESM.pdf]

Supplementary Information

Metabolomics Facilitates Differential Diagnosis in Common Inherited Retinal Degenerations by Exploring Their Profiles of Serum Metabolites

Wei-Chieh Wang, Chu-Hsuan Huang, Hsin-Hsiang Chung, Pei-Lung Chen, Fung-Rong Hu, Chang-Hao Yang, Chung-May Yang, Chao-Wen Lin, Cheng-Chih Hsu*, and Ta-Ching Chen*

Supplementary Table 1. Baseline characteristics of IRD patients and healthy participants.

Supplementary Table 2. Number of significant features in each comparison, which were used to quantify the metabolic differences between each IRD subtype and healthy controls.

Supplementary Table 3. The weighting and identification results for selected metabolites from the machine learning model for diagnosing (a) healthy participants, CD/CRD, and STGD, and (b) *EYS-*, *USH2A-*, and other RP.

Supplementary Figure 1. Violin plots of age-adjusted abundance of D-Xylonate (a), citronellyl acetate (b), hexadecanedioic acid (c), PS(14:1/16:0) (d), PC(19:1) (e), PC(16:0/9:0(CHO)) (f), and N-undecanoylglycine (g) in different IRD groups.

Supplementary Figure 2. Volcano plots of comparisons between each IRD subgroup and the control group (a – h), STGD and CDCRD (i), and RP with the ABCA4 genotype and STGD (j), after age adjustment.

Supplementary Figure 3. The violin plot and ROC curve of identified metabolites are selected using a machine learning model to diagnose (a) STGD and CD/CRD, and (b) EYS- and USH2A- associated RP.

Supporting information for method section

Supplementary Table 1. Baseline characteristics of IRD patients and healthy participants. A total of 155 participants were enrolled in the present study. Statistical analysis was performed using Chi-square/Fisher's exact test (two-sided) and analysis of variance (ANOVA) with Tukey's honestly significant difference test (two-sided).

Disease	Causative gene	Case Number	Sex		Age (mean ± SD)	Smoking habit	Systemic Disease	Anti-oxidant supplement	BMI (mean ± SD)
			Female	Male					
**Healthy participants**	-	28	17	11	43.4 ± 17.3	0	6, 21.4%	5, 17.9%	23.3 ± 3.3
**RP**	Overall	70	41	29	48.7 ± 14.3	2, 2.8%	14, 20.0%	60, 85.1%	23.1 ± 3.8
	EYS	25	12	13	48.6 ± 15.3	1, 4%	4, 16%	23, 92%	22.6 ± 3.3
	USH2A	23	15	8	48.2 ± 13.1	1, 4.3%	5, 21.7%	19, 82.6%	22.7 ± 3.8
	ABCA4	11	8	3	51.1 ± 15.4	0	3, 27.3%	9, 81.2%	23.6 ± 4.9
	PRPF31	11	6	5	47.3 ± 15.2	0	2, 18.2%	9, 81.2%	24.3 ± 4.0
**STGD**	*ABCA4*	20	12	8	29.0 ± 21.1	0	4, 20%	14, 70%	22.0 ± 5.5
**CD/CRD**	-	21	8	13	41.4 ± 14.4	0	6, 28.6%	17, 80.9%	22.5 ± 4.4
**BCD**	*CYP4V2*	16	12	4	49.2 ± 13.3	1, 6.3%	5, 31.3%	13, 81.3%	23.0 ± 2.5
p-value			0.352		0.001*	0.726	0.953	0.001#	0.895

BCD, Bietti's crystalline dystrophy; CD/CRD, cone dystrophy/cone-rod dystrophy; IRD, inherited retinal degeneration; RP, retinitis pigmentosa; STGD, Stargardt disease; SD, standard deviation

* post-Hoc analysis: STGD vs healthy participants, p=0.024; STGD vs RP (overall), p<0.001; STGD vs BCD, p=0.003.

# In all IRD groups, p=0.709.

Supplementary Table 2. Number of significant features in each comparison, which were used to quantify the metabolic differences between each IRD subtype and healthy controls

Comparison		Number of significant metabolic features		
**Group I**	**Group II**	**Up regulate**	**Down regulate**	**Total**
BCD	Normal	0	0	0
CD/CRD	Normal	70	190	260
STGD	Normal	72	75	147
RP (all genotype)	Normal	156	65	221
RP (*ABCA4*)	Normal	102	75	177
RP (*PRPF31*)	Normal	85	79	164
RP (*EYS*)	Normal	124	72	196
RP (*USH2A*)	Normal	123	41	164
CD/CRD	STGD	7	64	71
STGD	RP (*ABCA4*)	13	6	19

Significant features are defined as a false discovery rate < 0.05 (Benjamini–Hochberg test, two-sided) and fold change > 2.

BCD, Bietti's crystalline dystrophy; CD/CRD, cone dystrophy/cone-rod dystrophy; IRD, inherited retinal degeneration; RP, retinitis pigmentosa; STGD, Stargardt disease

Supplementary Table 3. The weighting and identification results for selected metabolites from the machine learning model for diagnosing (a) healthy participants, CD/CRD, and STGD, and (b) *EYS*-, *USH2A*-, and other RP.

Weighting	Observed *m/z*	Retention time (min)	Compound name	Chemical formula	Ion species	Mass error (ppm)
4.94	200.2010	5.79	Dodecanamide	$C_{12}H_{25}NO$	$[M+H]^+$	-2.19
4.77	287.2220	6.48	Hexadecanedioate	$C_{16}H_{30}O_4$	$[M+H]^+$	-0.89
4.00	244.1907	2.20	N-Undecanoylglycine	$C_{13}H_{25}NO_3$	$[M+H]^+$	-2.45
1.37	612.5569	7.51	Diacylglycerol (16:0/18:1)	$C_{37}H_{70}O_5$	$[M+NH_4]^+$	0.30
0.26	188.1758	0.97	N8-Acetylspermidine	$C_9H_{21}N_3O$	$[M+H]^+$	-2.43
**Weighting**	**Observed m/z**	**Retention time (min)**	**Compound name**	**Chemical formula**	**Ion species**	**Mass error (ppm)**
4.04	200.2010	5.79	Dodecanamide	$C_{12}H_{25}NO$	$[M+H]^+$	-2.19
3.55	425.2889	8.12	alpha-Phocaecholic acid	$C_{24}H_{40}O_6$	$[M+H]^+$	-3.30
3.32	275.1843	2.34	3-Hydroxytetradecanedioic acid	$C_{14}H_{26}O_5$	$[M+H]^+$	-5.63
3.30	297.2425	8.13	13-hydroxyoctadecadienoic acid	$C_{18}H_{32}O_3$	$[M+H]^+$	-1.72
3.16	857.7591	15.68	TG(18:1/16:0/18:2)	$C_{55}H_{100}O_6$	$[M+H]^+$	-0.85
2.75	256.2269	7.55	9-Decenoylcholine	$C_{15}H_{30}NO_2$	$[M]^+$	-2.83
2.63	162.1126	0.97	Carnitine	$C_7H_{16}NO_3$	$[M]^+$	-2.65
1.45	322.0794	1.03	Deoxy-5-methylcytidylate	$C_{10}H_{16}N_3O_7P$	$[M+H]^+$	-3.03
1.31	720.5895	10.78	PC(o32:0)	$C_{40}H_{82}NO_7P$	$[M+H]^+$	-1.63
1.29	793.6280	7.73	TG(46:4)	$C_{49}H_{86}O_6$	$[M+Na]^+$	-5.32
0.70	1047.7333	7.34	LysoPC(18:0)	$C_{26}H_{54}NO_7P$	$[2M+H]^+$	-2.03
0.62	796.5825	10.46	PC(37:4)	$C_{45}H_{82}NO_8P$	$[M+H]^+$	-3.91
0.46	901.7246	14.95	TG(18:2/18:2/20:5)	$C_{59}H_{96}O_6$	$[M+H]^+$	-4.33
0.33	818.6060	10.14	PC(40:6)	$C_{48}H_{84}NO_7P$	$[M+H]^+$	-0.50

CD/CRD, cone dystrophy/cone-rod dystrophy; IRD, inherited retinal degeneration; RP, retinitis pigmentosa; STGD, Stargardt disease; LysoPC, lysophosphatidylcholine; PC, phosphatidylcholine; TG, triglyceride.

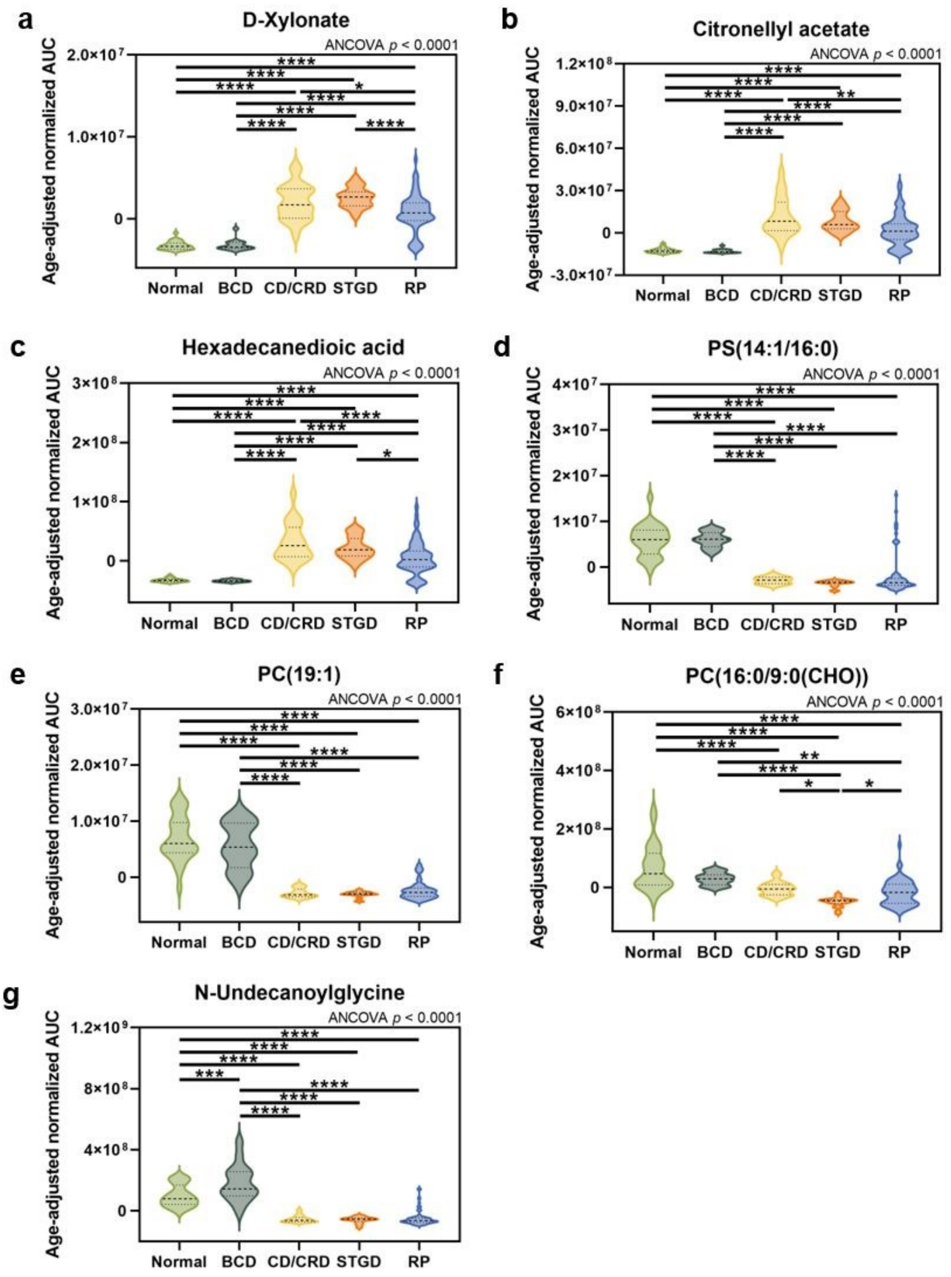

Supplementary Figure 1. Violin plots of age-adjusted abundance of D-Xylonate (a), citronellyl acetate (b), hexadecanedioic acid (c), PS(14:1/16:0) (d), PC(19:1) (e), PC(16:0/9:0(CHO)) (f), and N-undecanoylglycine (g) in different IRD groups. The analysis of covariance (ANCOVA) was utilized to adjust metabolite abundance while controlling for the age covariate through linear regression, followed by post hoc Tukey's test.

IRD, inherited retinal degeneration; BCD, Bietti's crystalline dystrophy; CRD, cone-rod dystrophy; RP, retinitis pigmentosa; STGD, Stargardt disease;

ns: $p > 0.05$; *: $p \leq 0.05$; **: $p \leq 0.01$; ***: $p \leq 0.001$; ****: $p \leq 0.0001$.

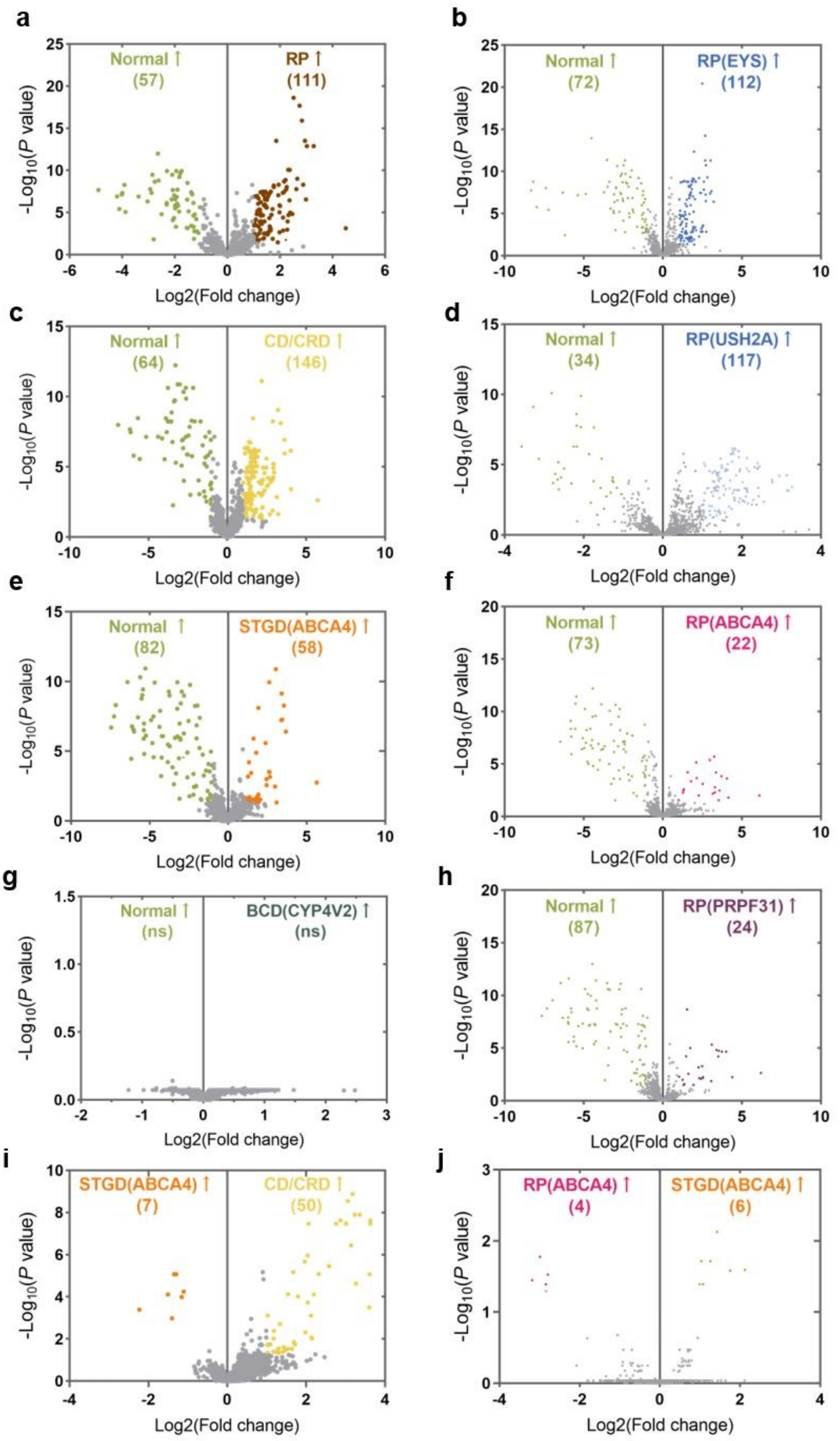

Supplementary Figure 2. Volcano plots of comparisons between each IRD subgroup and the control group (a – h), STGD and CDCRD (i), and RP with the ABCA4 genotype and STGD (j), after age adjustment. The analysis of covariance (ANCOVA) was utilized to adjust metabolite abundance while controlling for the age covariate through linear

regression. Significant features in the volcano plot were defied as a false discovery rate < 0.05 (Benjamini-Hochberg test) and fold change > 2.

IRD, inherited retinal degeneration; BCD, Bietti's crystalline dystrophy; CRD, cone-rod dystrophy; RP, retinitis pigmentosa; STGD, Stargardt disease.

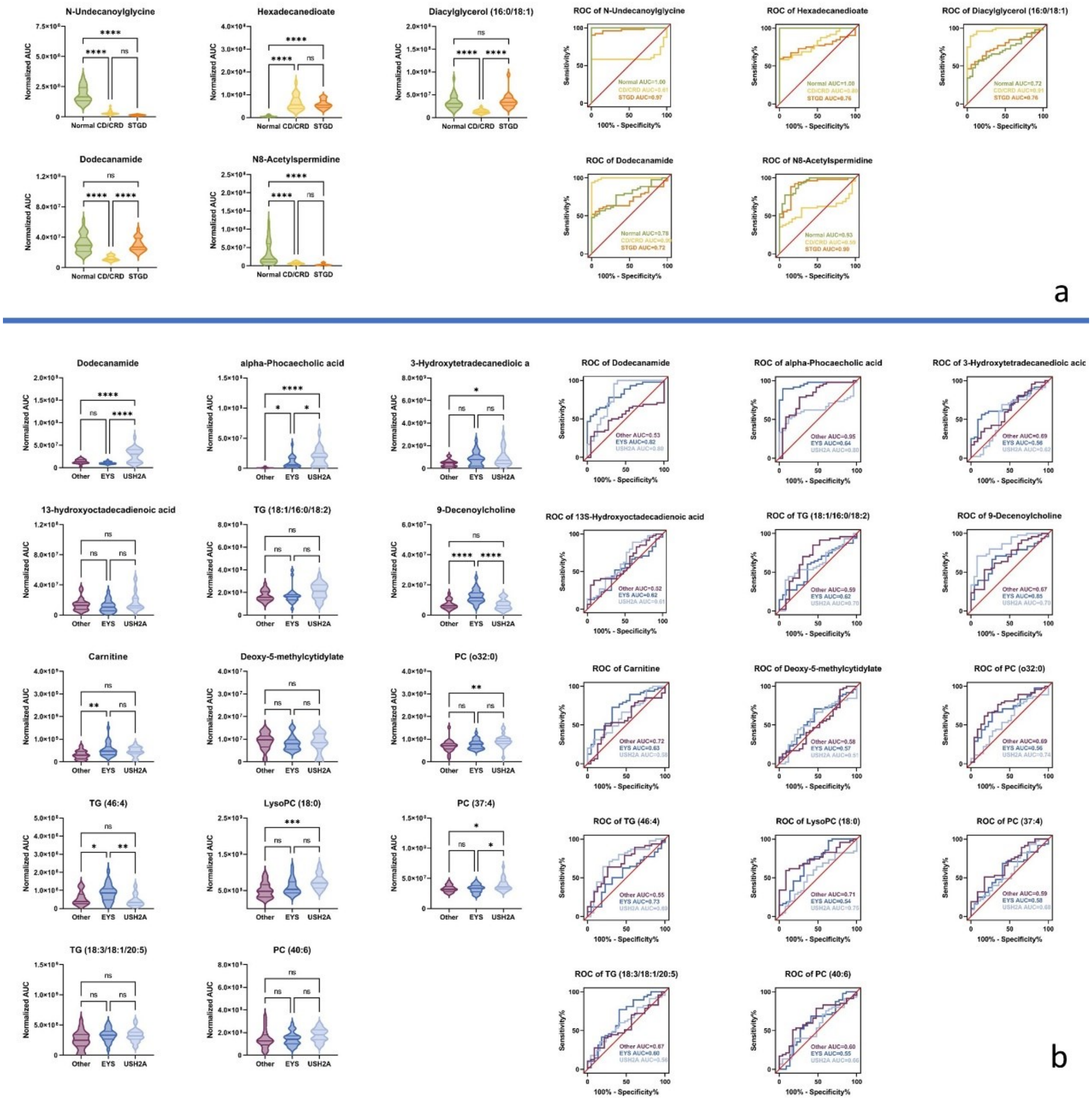

Supplementary Figure 3.

(a) The violin plot and ROC curve of five identified metabolites are selected using a machine learning model to diagnose STGD and CD/CRD.

(b) The violin plot and ROC curve of 14 identified metabolites are selected using a machine learning model to diagnose EYS- and USH2A- associated RP.

ROC, receiver operating characteristic curve; CD/CRD, cone dystrophy/cone-rod dystrophy; STGD, Stargardt disease; RP, retinitis pigmentosa

Supporting information for method section

Chemicals and reagents

Methyl tert-butyl ether (MTBE) was purchased from Thermo Fisher Scientific (Extra Pure grade; MA, US). Ammonium acetate (AA), L-tryptophan-(indole-d5), and isopropanol (IPA) were purchased from Sigma-Aldrich (MO, USA). Acetonitrile (ACN) and methanol (MeOH) were purchased from J.T. Baker (LCMS grade, NJ, USA). Formic acid (FA) was purchased from Honeywell (98% purity, NJ, USA). Chemical standards, including 1-pentadecanoyl-2-oleoyl(*d*7)-sn-glycero-3-phosphocholine (15:0-18:1-*d*7-PC) and 1-pentadecanoyl-2-oleoyl(*d*7)-sn-glycero-[phosphorpho-rac-(1'-glycerol)] (15:0-18:1-*d*7-PG) were purchased from Avanti Polar Lipids (Alabaster, AL, USA). Ultrapure water was obtained using PureLab Classic (ELGA, UK).

Serum metabolites extraction

The MTBE extraction protocol, with laboratory modifications, was used to extract lipids and polar metabolites from the serum. The 10 µL internal standards (IS) mixture contains 15:0-18:1-d7-PC (2 ppm), 15:0-18:1-d7-PG (2 ppm), and L-tryptophan-(indole-d5) (10 ppm) were spiked into an aliquot of 50 µL serum. Then the sample was extracted by adding 600 µL MTBE and 150 µL MeOH and vortexed for 30 min at room temperature.

Next, the sample was added with 200 µL water and centrifuged for 3 min at 13,697 g for phase separation. The upper portion containing serum lipids was transferred to another tube. The extraction was repeated by adding 100 µL water, 100 µL MeOH, and 300 µL MTBE. The sample was vortexed for an additional 10 min and centrifuged for 3 min at 13,697 g. The upper portion was mixed with the lower portion, and the combined solution was dried in a vacuum concentrator (Vacufuge plus Vacuum Concentrator, Eppendorf) for 3 h. The sample reconstitution was performed by adding 100 µL of reconstituted solution (ACN/IPA/water, v/v/v = 65/30/5).

For the lower portion, 150 µL cold MeOH was added and stored under a -20 ^{o}C environment for 2 h, followed by 10 min of 21,401 g centrifugation for protein precipitation. Next, the supernatant was dried using a vacuum concentrator overnight and then reconstituted by adding 100 µL reconstituted solution (ACN/water, v/v = 50/50). The protein precipitation was repeated by mixing 60 µL reconstituted sample with 120 µL cold ACN and then putting the mixtures in a -20 ^{o}C freezer for an hour. After a 15 min centrifugation at 21,401 g under 4 ^{o}C, super supernatants (120 µL) were collected and stored under -80 ^{o}C before further analysis.

LC-MS analysis

Untargeted metabolomic analyses rely on ultra-performance liquid chromatography-tandem mass spectrometry (UPLC-MS/MS). The platform utilized a Thermo Scientific UltiMate 3000 UHPLC system and Q Exactive Plus Hybrid Quadrupole-Orbitrap MS interfaced with a heated electrospray ionization probe. A 5 µL volume of upper (hydrophobic) and lower (hydrophilic) portion serum extract was separated using a C18 (Waters UPLC CSH C18: 2.1 x 100 mm, 1.7 µm) and a hydrophilic interaction liquid chromatography (HILIC) column (Waters UPLC BEH Amide: 2.1 x 150 mm, 1.7 µm), respectively. The MS scan range covered 150–1,500 and 70–1,000 *m/z* for the hydrophobic and hydrophilic portion analyses, respectively. Each portion was analyzed under positive and negative ion modes; the spray voltage is 3.5 and -3.5 kV, respectively. The MS operated at 180 °C heater temperature, 280 °C capillary temperature, 35 arb sheath gas flow, and 15 arb auxiliary gas flow. The Orbitrap mass analyzer was set at 35,000 mass resolution.

The injection order of the samples has been randomly arranged for the LC-MS analysis, and pooled quality control samples were injected at the beginning, every 10 injection, and at the end of each batch to ensure spectral quality.

The upper portion of the extract containing the relatively hydrophobic metabolites was gradient-eluted using IPA, ACN, and water (A: ACN/H2O, v/v = 4/6; B: IPA/ACN, v/v = 9/1), each containing 0.1% FA and five mM AA for positive and negative ion mode scanning, respectively. The UPLC elution conditions for positive ion mode were: 0–1 min, 15% B; 1–15 min, 15–70% B; 15-16 min, 70–99% B; 16–18.5 min, 99% B; 18.5–19 min, 99–15% B; and 19-–21 min, 15% B. The gradients for negative ion mode were: 0–0.5 min, 15% B; 0.5–4 min, 15–70% B; 4–15 min, 70–99% B; 15–16.5 min, 99% B; 16.15–17 min, 99–15% B; and 17–19 min, 15% B. The flow rate and column temperature were set to 0.2 mL/min and 50 ^{o}C.

The lower portion of the extract containing relatively hydrophilic compounds was gradient-eluted using water (A) and ACN (B) containing 0.1% FA and five mM AA for positive and negative ion mode analyses, respectively. The optimized UPLC elution conditions were: 0–1 min, 90% B; 1–9 min, 90-40% B; 9–11.5 min, 40% B; 11.5–12 min, 40–90% B; and 12–14 min, 90% B. The flow rate and column temperature were set to 0.25 mL/min and 40 ^{o}C.

Data preprocessing

UPLC-MS/MS data were processed using Thermo Scientific Compound Discoverer v3.2 software. Based on the cubic-line retention time alignment algorithm, the retention times were aligned to the references selected using the software. Next, only features with intensities above 1,000,000 and 500,000 for the positive and negative datasets, respectively, were included for further analysis. M+H, M+Na, M+K, M+H-H_2O,

$M+H-NH_3$, $M+2H$, $M-H$, $M-H-H_2O$, and $M-2H$ were considered possible adduct ions, and each ion was assigned to a predicted chemical composition according to its exact mass (< 5 ppm) and isotopic pattern (intensity tolerance < 30%). Different adduct ions from the same compound were grouped, and only metabolites that appeared in at least half of the samples in any IRD subtype or healthy controls were preserved. Undetectable metabolites in the samples were filled using background noise. The signal of each metabolite was normalized to the corresponding IS for each dataset. The references for *hydrophobic-positive*, *hydrophobic-negative*, and *hydrophilic-(both positive and negative)* were 15:0-18:1-d7-PC, 15:0-18:1-d7-PG, and L-tryptophan-(indole-d5), respectively. Finally, the peak intensity lower than three times blank samples were excluded, and 1,606 compounds were received after the above processing.

Statistical analysis

Next, multivariate and univariate statistical analyses were conducted using the MetaboAnalyst 5.0 online platform.[41] The heatmap was constructed based on the auto-scale features standardization, Pearson distance measurement, and Ward clustering method. Partial least squares-discriminant analysis was performed on normally distributed data. The significant features in the volcano plot were defined as a false discovery rate < 0.05 (Benjamini–Hochberg test) and fold change > 2.

Machine learning and diagnostic model construction

The least absolute shrinkage and selection operator (LASSO) algorithm was applied to establish two diagnostic models: one for CD/CRD, STGD, and normal participant classification, and the other for RP patients' genotype prediction. The RP genotyping model discriminated between USH2A, EYS, and other samples, including ABCA4 and PRPF31 mutants. In this study, RapidMiner Studio (version 9.10.001), a commercially available data mining software, was used to construct the machine learning models. To implement the LASSO model, the built-in generalized linear model was used and the alpha parameter was set to 1, indicating the use of an L1 penalty. The lambda parameter, controlling the degree of regularization, was determined using the "lambda search" function within RapidMiner. The optimization process programmed to terminate when the relative improvement fell below 0.001. The MS data were randomly separated into training and validation sets with a ratio of 7 to 3 for each subtype. The LASSO models were trained using the training dataset, and evaluated using leave-one-out cross-validation. After training, the model was further evaluated using the validation set, and the area under the ROC curve was calculated for the training and validation dataset, respectively.

Compound identification

The *m/z* features selected using the machine learning diagnostic model or potential biomarkers revealed using the statistical analysis were determined using the exact mass and MS/MS spectrum. The confidence level of metabolite assignment is according to the guidelines reported by the Chemical Analysis Working Group of the Metabolomics Standards Initiative.[42] There are four confidence levels for metabolite identification, where level one represents identified compounds, and level four represents unknown compounds. In this study, the features were labeled as level two if the error of the exact mass was less than 5 ppm and the MS/MS spectrum was interpretable or similar to the databases. The confidence level is labeled as three if the identification is based only on a faithful group.
